# Conventional cardiopulmonary resuscitation-induced refractory cardiac arrest due to latent left ventricular outflow tract obstruction due to a sigmoid septum: a case report

**DOI:** 10.1186/s13256-018-1767-z

**Published:** 2018-08-20

**Authors:** Toshinobu Yamagishi, Takahiro Tanabe, Hiroshi Fujita, Kazuki Miyazaki, Takahiro Yukawa, Kazuhiro Sugiyama, Yuichi Hamabe

**Affiliations:** 1 Tertiary Emergency Medical Center, Tokyo Metropolitan Bokutoh Hospital, 4-23-15 Kotobashi, Sumida-ku, Tokyo, 130-8575 Japan; 2 Department of Transfusion Medicine, Tokyo Metropolitan Bokutoh Hospital, Tokyo, Japan

**Keywords:** Left ventricular outflow tract obstruction, Heart arrest, Cardiopulmonary resuscitation, Hemolytic anemia, Adrenaline

## Abstract

**Background:**

Patients with left ventricular outflow tract obstruction who do not exhibit a dynamic pressure gradient at rest, experience pressure gradient increases of ≥ 30 mmHg only during specific situations; this is called latent left ventricular outflow tract obstruction. It is provoked by increased cardiac contraction and preload and afterload depletion. There are a few reports of patients with it developing cardiac arrest. We present a case of latent left ventricular outflow tract obstruction in which the patient with a sigmoid septum experienced refractory pulseless electrical activity due to conventional advanced cardiac life support.

**Case presentation:**

A 73-year-old Asian woman on escitalopram and lorazepam was transported to our hospital for chest and back pain with altered consciousness. On arrival, she was in shock and developed pulseless electrical activity. After initiation of conventional cardiopulmonary resuscitation according to adult advanced cardiovascular life support guidelines, she could not regain spontaneous circulation. She was ultimately resuscitated via venoarterial extracorporeal membrane oxygenation initiation. The only abnormal laboratory result at admission was anemia. Her hemodynamic status stabilized after red blood cell transfusion, and venoarterial extracorporeal membrane oxygenation was subsequently terminated. Transthoracic echocardiography showed a sigmoid septum; dobutamine-infused Doppler echocardiography revealed a significant outflow gradient, and continuous monitoring showed Brockenbrough–Braunwald sign, which confirmed a diagnosis of latent left ventricular outflow tract obstruction due to a sigmoid septum. As a result, carvedilol and verapamil were initiated. A follow-up dobutamine-infused Doppler echocardiography showed a reduction of outflow gradient, and she was discharged without any sequelae. Latent left ventricular outflow tract obstruction worsened due to increasing cardiac contraction and the depletion of preload and afterload. Depleted preload occurred due to dehydration and anemia, whereas depleted afterload occurred due to the prescribed drugs, which subsequently caused pulseless electrical activity. Moreover, β-stimulation from the adrenaline probably enhanced the hypercontractile state and caused refractory pulseless electrical activity in our case.

**Conclusions:**

Patients with latent left ventricular outflow tract obstruction can progress to cardiogenic shock and pulseless electrical activity due to increased cardiac contraction and depletion of preload and afterload. We should consider the patient’s underlying conditions that induced pulseless electrical activity.

## Background

Left ventricular outflow tract obstruction (LVOTO) influences the symptom progression and death of patients with hypertrophic cardiomyopathy (HCM) [1, 2] and a sigmoid-shaped ventricular septum [3, 4]. LVOTO is defined as a left ventricular outflow tract pressure gradient increase of ≥ 30 mmHg [3], and approximately 25% of patients with HCM have dynamic LVOTO at rest [1, 2]. Conversely, those patients with HCM who do not exhibit dynamic pressure gradient at rest experience pressure gradient increases of ≥ 30 mmHg during specific situations only, which is called latent LVOTO [5]. Latent LVOTO is provoked by increased cardiac contraction and preload and afterload depletion [6, 7].

There have been few reports of patients with latent LVOTO developing cardiac arrest. We present a case of a patient with a sigmoid septum who experienced cardiac arrest due to latent LVOTO, as well as refractory pulseless electrical activity (PEA) due to conventional cardiopulmonary resuscitation (CPR).

## Case presentation

**Fig. 1:**
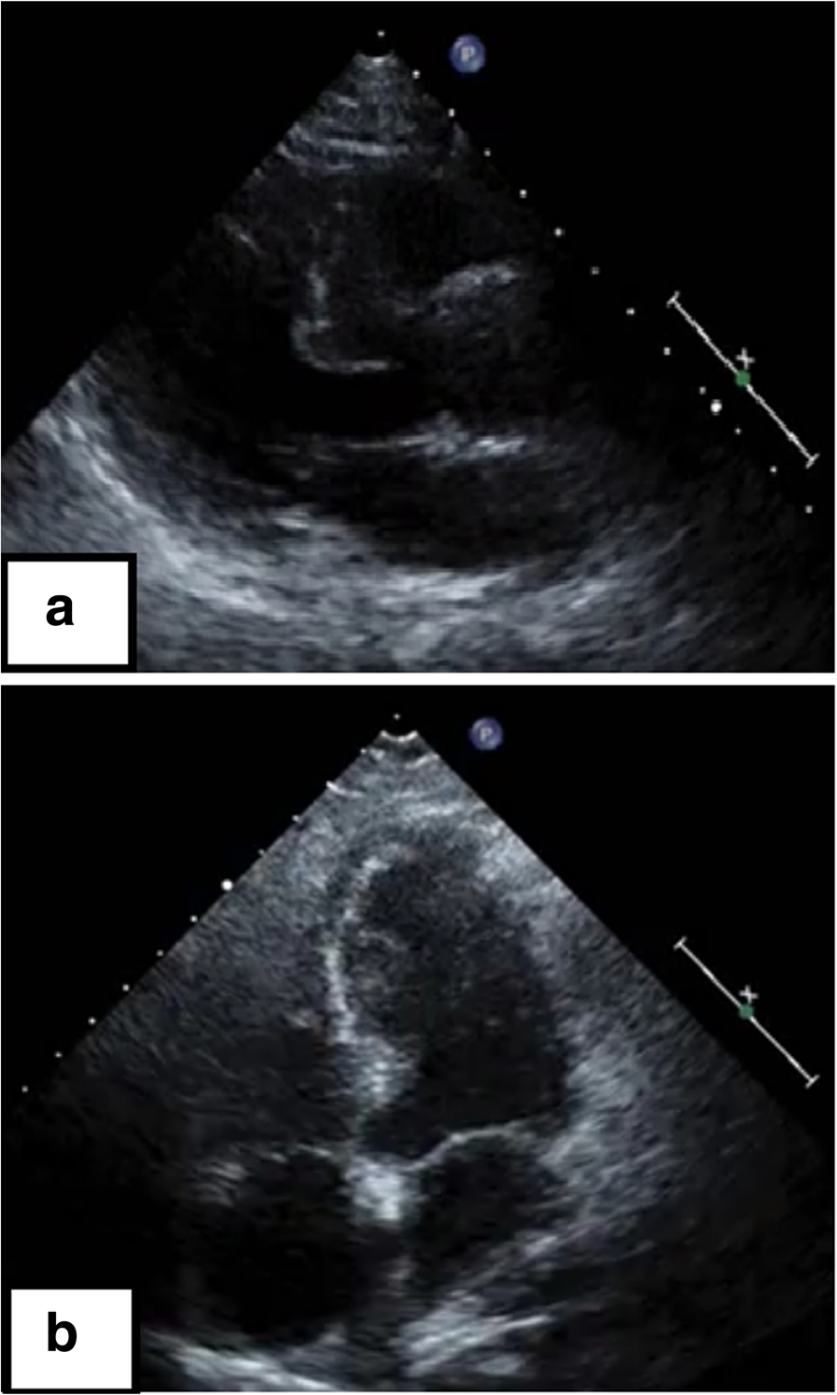
Transthoracic echocardiography on day 10. In the parasternal long axis view (**a**) and four-chamber view (**b**), echocardiography revealed a morphological characteristic of the basal interventricular septum that protrudes into the left ventricular cavity

**Fig. 2:**
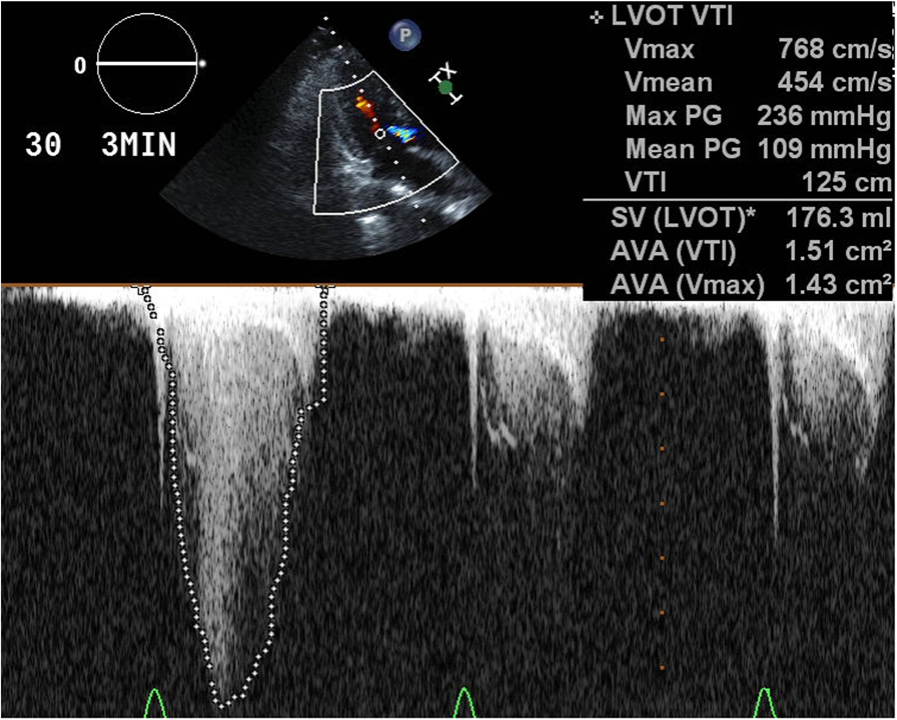
Provocation with dobutamine infusion (30 μg/kg per minute) during Doppler echocardiography on day 26. Echocardiography reveals a left ventricular outflow gradient of 236 mmHg, which confirms a diagnosis of latent left ventricular outflow tract obstruction. *LVOT* left ventricular outflow tract

**Fig. 3:**
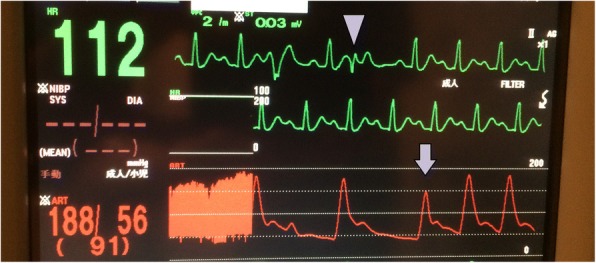
Brockenbrough–Braunwald sign during dobutamine-infused (30 μg/kg per minute) Doppler echocardiography on day 26. Continuous electrocardiogram and invasive radial arterial blood pressure show a Brockenbrough–Braunwald sign, which is a fall of arterial blood pressure (*arrow*) after a premature ventricular contraction (*arrowhead*)

A 73-year-old Asian woman with an underlying anxiety disorder, functional headache, and hypertension was prescribed escitalopram and lorazepam when she presented with progressively worsening headaches to her primary care doctor. Her symptoms did not improve with the medications, and she was unable to eat well and required bed rest. She was transported to our hospital 4 days later after developing chest and back pain with altered consciousness. She was a housekeeper, had no allergies, and had no alcohol or tobacco smoking history. On arrival, her Glasgow Coma Scale score was 3/15 (E1V1M1); both pupils were approximately 4 mm in diameter and reactive. Her blood pressure was too low to be measured, her carotid artery pulse was palpable, her heart rate was 112 beats/minute, and her respiratory rate was 30 breaths/minute. Her conjunctiva was pale. An auscultation of breath sounds did not reveal upper and lower airway obstructions and was within normal limits. Her abdomen was soft and flat without tenderness. She had no skin abnormalities (such as rash). Both legs had no edema. Echocardiography on arrival was performed as point of care ultrasound and revealed a hypercontractile left ventricle with an eliminated left ventricular cavity and a collapsed inferior vena cava without right ventricular dilation. There was no pericardial effusion or obvious large regurgitant jet observed on color Doppler. In response, we immediately inserted a peripheral venous catheter and began introducing fluid resuscitation; however, she developed PEA. Conventional CPR according to the adult advanced cardiovascular life support guidelines (including adrenaline) was initiated and a return of spontaneous circulation (ROSC) occurred. However, her blood pressure was unstable and PEA returned, prompting repeated CPR with immediate administration of fluids and three adrenaline injections. Venoarterial extracorporeal membrane oxygenation (VA-ECMO) was initiated for refractory PEA. Whole-body contrast-enhanced computed tomography was unremarkable, and the admission laboratory results were also unremarkable, except for anemia (Table 1). Her hemoglobin level decreased from 7.1 g/dL to 3.5 g/dL 1 hour later without obvious signs of gastrointestinal hemorrhage. Therefore, 8 units of packed red blood cells were transfused for 1 day, after which her hemodynamic status stabilized. She was in a coma without sedatives; thus, targeted temperature management at 34 °C was initiated on admission to an intensive care unit. Echocardiography in the intensive care unit showed a thickened interventricular septum (which was 12.8 mm), prolonged anterior mitral valve, and contact between the bodies of the anterior and posterior mitral valves, suggesting that the left ventricular obstruction could have potentially occurred through this redundant anterior mitral valve. VA-ECMO was terminated on day 3, and after stabilizing her hemodynamics, transthoracic echocardiography showed a sigmoid septum with normal left ventricular function (ejection fraction, 75%) (Fig. 1). On day 26, dobutamine-infused (30 μg/kg per minute) Doppler echocardiography revealed a significant outflow gradient (236 mmHg) accompanied with chest pain (Fig. 2) and intermittent systolic anterior motion (SAM) of the mitral valve; continuous monitoring during Doppler echocardiography showed a Brockenbrough–Braunwald sign (Fig. 3), which is a fall of arterial blood pressure after premature ventricular contraction; these findings confirmed a diagnosis of latent LVOTO due to a sigmoid septum. The significant LVOTO was not dependent on SAM but might have occurred due to the greatly thickened interventricular septum. As a result, carvedilol was initiated with gradual increment up to 10 mg/day on day 35. In addition, verapamil (120 mg/day) was administered on day 29. A follow-up dobutamine-infused Doppler echocardiography on day 40 showed a reduction of the outflow gradient to 14 mmHg, indicating a successful medical therapy.

**Table 1:** Laboratory data on day 1

	Patient’s result	Reference ranges
Complete blood count		
White blood cell count (/μL)	11,400	2700–10,300
Red blood cell count (× 10,000/μL)	254	357–497
Hemoglobin (g/dL)	7.1	11.4–14.2
Hematocrit (%)	20.7	32.3–43.1
Reticulocyte count (%)	13.4	0.2–2.7
Platelet (× 10,000/μL)	24.8	13.0–35.0
Coagulation test		
Prothrombin time (%)	97.6	75–120
International normalized ratio of prothrombin time (seconds)	0.99	
Activated partial thromboplastin time (seconds)	24.3	24–39
Biochemical test		
Total protein (g/dL)	6.3	6.0–8.3
Albumin (g/dL)	3.6	3.6–5.1
Blood urea nitrogen (mg/dL)	12	7.0–20.0
Creatinine (mg/dL)	0.8	0.4–0.9
Total bilirubin (mg/dL)	3.1	0.2–1.1
Direct bilirubin (mg/dL)	0.7	< 0.4
Sodium (mEq/L)	135	135–147
Potassium (mEq/L)	3.8	3.6–5.1
Chlorine (mEq/L)	100	98–108
Creatine kinase (U/L)	99	30–150
Creatine kinase-MB (U/L)	11	< 20
Aspartate aminotransferase (U/L)	79	10–37
Alanine aminotransferase (U/L)	45	6–35
Lactate dehydrogenase (U/L)	833	115–250
Alkaline phosphatase (U/L)	240	122–378
Gamma-glutamyl transpeptidase (U/L)	21	1–40
Iron (μg/dL)	143	55–180
Total iron binding capacity (μg/dL)	132	130–320
Unsaturated iron binding capacity (μg/dL)	275	260–420
Glucose (mg/dL)	391	60–110
Hemoglobin A1c (%)	5.1	4.6–6.2
Brain natriuretic peptide (pg/mL)	81.7	0–18.4
Blood gas analysis (10 L/minute O_2_ given)		
pH	7.105	7.35–7.45
PaO_2_ (mmHg)	129	≧ 80
PaCO_2_ (mmHg)	37.8	35–45
Hydrogen-carbonate ion (mmol/L)	11.4	22–26
Lactate (mmmol/L)	12.9	0.56–1.39
Anion gap (mmol/L)	27.6	20–26

*PaCO*_*2*_ partial pressure of carbon dioxide in arterial blood, *PaO*_*2*_ partial pressure of oxygen in arterial blood

The worsening anemia that was identified at admission was suspected to be hemolytic anemia (HA) based on results of the blood test. The results of the laboratory examination, including total bilirubin, direct bilirubin, lactate dehydrogenase, reticulocyte count, and haptoglobin, and a direct Coombs test on day 1 are presented in Table 1. Her drug lymphocyte stimulation test was positive, and agglutination occurred when her serum reacted with lorazepam. Therefore, lorazepam-induced immunological HA was diagnosed. Her anemia improved following discontinuation of lorazepam. Her hemoglobin level was 11.9 g/dL on day 64, and she was eventually discharged on day 68 without any sequelae.

## Discussion

Our patient developed refractory PEA that was managed with fluid resuscitation and blood transfusion via VA-ECMO initiation. She was diagnosed as having latent LVOTO due to a sigmoid septum. A sigmoid-shaped septum is generally considered part of the normal ageing process, but some cases showed latent LVOTO in which the gradient of LVOTO increased due to increased cardiac contraction and depleted preload and afterload, which leads to symptom deterioration [3, 6]. Previous studies on latent LVOTO reported that refractory cardiogenic shock and PEA were secondary to exercise and dehydration due to diarrhea [8, 9]. Here we consider the important clinical findings from this case in relation to latent LVOTO. In particular, the incidence of hemodynamic instability and refractory PEA was probably influenced by drug-induced immunological HA, dehydration, antidepressant drug use, and adrenaline administration.

Drug-induced immunological HA and dehydration might induce significant depletion of preload, influence the hypercontractile state, and contribute to dynamic LVOTO and shock in patients with latent LVOTO. The use of benzodiazepines and selective serotonin reuptake inhibitors may reduce afterload. Benzodiazepines inhibit the sodium, potassium, and calcium channels in the synapses. Serotonin reuptake inhibitors also appear to inhibit calcium channels, leading to vasodilation [10]. Therefore, in our case, escitalopram and lorazepam may have caused depletion of afterload and led to the harmful effect of increasing the gradient of LVOTO. The 2015 adult advanced cardiovascular life support guidelines for CPR indicate that it may be reasonable to administer adrenaline as soon as feasible after the onset of cardiopulmonary arrest via a nonshockable rhythm [11]; however, β-stimulation from the adrenaline injection might have enhanced the hypercontractile state. This harmful side effect may outweigh the potential benefit of α-stimulation (which increases the afterload), and it triggered refractory PEA in our case because cardiac output and systemic vascular resistance determine the blood pressure. The treatment for cardiogenic shock due to LVOTO consists of both β-blockers and vasoconstrictors, such as phenylephrine, metaraminol, and norepinephrine [5, 12]. PEA can be successfully treated if the conditions are identified and corrected [11]; hence, we should consider the patient’s underlying conditions that initially induced PEA and should remain cognizant that adrenaline could induce refractory PEA, similar to our case.

We also considered distributive shock, such as a systemic anaphylactic state or sepsis, or obstructive shock, such as cardiac tamponade or tension pneumothorax, upon our patient’s arrival. However, both clinical situations were not confirmed on physical examination, echocardiography, and enhanced computed tomography. Therefore, it is reasonable to consider that refractory PEA occurred in relation to latent LVOTO.

## Conclusions

Patients with latent LVOTO due to a sigmoid septum could progress to cardiogenic shock and PEA due to increased cardiac contraction and depletion of preload and afterload. We should consider the patient’s underlying conditions that induced PEA and remain aware that such patients could experience refractory PEA due to conventional CPR with adrenaline.

## Abbreviations

CPR: Cardiopulmonary resuscitation
HA: Hemolytic anemia
HCM: Hypertrophic cardiomyopathy
LVOTO: Left ventricular outflow tract obstruction
PEA: Pulseless electrical activity
ROSC: Return of spontaneous circulation
SAM: Systolic anterior motion
VA-ECMO: Venoarterial extracorporeal membrane oxygenation
